# Benefits of population‐level interventions for dementia risk factors: an economic modelling study

**DOI:** 10.1002/alz.085380

**Published:** 2025-01-09

**Authors:** Naaheed Mukadam, Robert Anderson, Sebastian Walsh, Raphael D Wittenberg, Martin Richard John Knapp, Carol Brayne, Gill Livingston

**Affiliations:** ^1^ University College London, London United Kingdom; ^2^ Care Policy and Evaluation Centre, London School of Economics and Political Science, London, Great London United Kingdom; ^3^ Cambridge Public Health, Cambridge United Kingdom; ^4^ Care Policy and Evaluation Centre (CPEC), London School of Economics, London, Greater London United Kingdom; ^5^ Care Policy and Evaluation Centre (CPEC), London School of Economics and Political Science, London United Kingdom; ^6^ Cambridge Public Health, University of Cambridge, Cambridge United Kingdom; ^7^ Camden and Islington NHS Foundation Trust, London United Kingdom

## Abstract

**Background:**

Some individual‐level interventions for dementia risk factors could be cost saving. We aimed to estimate the cost effectiveness of population‐level interventions for tackling dementia risk factors. We found such interventions for tobacco smoking, excess alcohol use, diet modification to decrease hypertension and obesity, air pollution, and head injury.

**Method:**

We used published intervention effect sizes and relative risks for each risk factor. We used a Markov model to estimate progression to dementia in populations with and without the intervention, looking at lifetime risk, using the population of England as an exemplar.

**Result:**

Based on the effects on dementia, we estimated that reductions in excess alcohol use through minimum unit pricing would be expected to produce cost savings of £280 million and 4767 QALY gains respectively. Reformulation of food products to reduce salt and sugar intake and therefore reduce obesity and hypertension would be expected to produce cost savings of £2.4 and £1.1 billion, and QALY gains of 39,433 and 17,985, respectively. Introducing low emission zones in the largest English cities without them, so decreasing pollution and dementia would be expected to lead to £260 million savings and 5119 QALYs gained. Raising cigarette prices by 10% to reduce smoking rates, would be expected to lead to 2277 QALY gains and savings of £157 million from its effect on dementia. Legislating for compulsory bicycle helmets for children reducing head injury and dementia would lead to 1554 QALY gains and £91 million savings.

**Conclusion:**

We advocate for these population‐level interventions to help tackle lifecourse dementia risk and be cost‐saving.

